# Genome-Wide Identification and Analysis of *SRO* Gene Family in Chinese Cabbage (*Brassica rapa* L.)

**DOI:** 10.3390/plants9091235

**Published:** 2020-09-18

**Authors:** Yali Qiao, Xueqin Gao, Zeci Liu, Yue Wu, Linli Hu, Jihua Yu

**Affiliations:** 1 College of Horticulture, Gansu Agricultural University, Lanzhou 730070, China; qiaoyl@st.gsau.edu.cn (Y.Q.); gaoxq@st.gsau.edu.cn (X.G.); liuzc@gsau.edu.cn (Z.L.); wuy@gsau.edu.cn (Y.W.); 2 Gansu Provincial Key Laboratory of Aridland Crop Science, Gansu Agricultural University, Lanzhou 730070, China

**Keywords:** Chinese cabbage, *SRO* gene family, abiotic stress, bioinformatics, expression analysis

## Abstract

Similar to radical-induced cell death 1 (SROs) is a family of small proteins unique to plants. *SRO* transcription factors play an important role in plants’ response to biotic and abiotic stresses. In this study, we identified 12 *BrSRO* genes in Chinese cabbage (*Brassica rapa* L.). Among them, a comprehensive overview of the *SRO* gene family is presented, including physical and chemical characteristics, chromosome locations, phylogenetic analysis, gene structures, motif analysis, and cis-element analyses. The number of amino acids of *BrSRO* genes is between 77–779 aa, isoelectric point changed from 6.02 to 9.6. Of the 12 *BrSRO* genes, 11 were randomly distributed along the 7 chromosomes, while *BrSRO12* was located along unassigned scaffolds. Phylogenetic analysis indicated that the SRO proteins from six species, including *Arabidopsis*, banana, rice, *Solanum lycopersicum*, *Zea mays*, and Chinese cabbage were divided into eleven groups. The exon-rich *BrSRO6* and *BrSRO12* containing 15 exons were clustered to group K. All 12 genes have motif 2, which indicate that motif 2 is a relatively conservative motif. There are many hormone and stress response elements in *BrSRO* genes. The relative expression levels of 12 *BrSRO* genes under high temperature, drought, salt, and low temperature conditions were analyzed by real-time fluorescence quantitative PCR. The results indicated the relative expression level of *BrSRO8* was significantly up-regulated when plants were exposed to high temperature. The relative expression levels of *BrSRO1*, *3*, *7*, *8*, and *9* were higher under low temperature treatment. The up-regulated genes response to drought and salt stresses were *BrSRO1*, *5*, *9* and *BrSRO1*, *8*, respectively. These results indicated that these genes have certain responses to different abiotic stresses. This work has provided a foundation for further functional analyses of *SRO* genes in Chinese cabbage.

## 1. Introduction

When subjected to stresses, plants can survive in complex and diverse environments for stress-induced gene expression. In these processes, similar to radical-induced cell death 1 proteins (SROs) participate in multiple regulatory networks through stress response [1,2]. *SRO* is a family of small proteins unique to plants. It plays an important role in plant growth and development and in responding to abiotic stresses, such as salt, drought, and heavy metals. *SROs* generally contain a poly(ADP-ribose) polymerase catalytic (PARP, PS51059) center and a RCD1-SRO-TAF4(RST, PF12174) conservative domain [3], part of the *SROs* also contains N-terminal WWE (PS50918) domain [4]. In *Arabidopsis*, there are six members in the *AtSRO* family, namely *AtRCD1* and *AtSRO1-5* [5]. AtRCD1 is the first member of the *SRO* family identified in *Arabidopsis* [6]. *AtRCD1* can interact with transcription factors in the nucleus to participate in the drought response mediated by the plant abscisic acid signaling pathway, and can also regulate plant development through hormone signaling pathways including abscisic acid (ABA), ethylene (ETH), methyl jasmonate (MEJA) and so on [7,8]. *AtSRO1* and *AtRCD1*, two homologous genes, have functional redundancy under different stress conditions [9]. *AtSRO1* is involved in abiotic stress response, and its mutant *SRO1-1* has strong resistance to osmotic and oxidative stress [3,10]. *AtSRO5* interacts with transcription factors to regulate gene expression, and overexpression of *AtSRO5* can increase the salt tolerance of transgenic plants by lowering the level of H_2_O_2_ in the roots [11]. *AtSRO2* and *AtSRO3* can respond to strong light, salt, and ozone stress; *AtSRO4* has no clear function reported [12]. *SROs* have also been partially studied in apple, rice, wheat, corn, continental cotton, tomato, and other crops. For example, in apple, *MdRCD1* can regulate the pore size through ABA signaling pathway, tolerate drought stress, and regulate root growth [13]. In rice, *OsSRO1c* participates in drought and oxidative stress through promoting stomatal closure and H_2_O_2_ accumulation by regulating SNAC1 and DST [14]. In wheat, *Ta-SRO1* can improve drought tolerance by regulating REDOX balance in plants [1]. The *SRO* gene families in various species have been identified, and the mechanism of *SROs* in response to drought stress is becoming increasingly clear.

Although a large number of studies on *SRO* genes in various species have been conducted, studies on *SRO* genes of Chinese cabbage have still not been reported. Chinese cabbage (*Brassica rapa* L.), which originated from China, is one of the specialty vegetables in the country. Chinese cabbage is rich in a variety of nutrients and is loved by consumers. Leaf bulb is the main edible part of Chinese cabbage. The growth and development of each organ of Chinese cabbage directly affect the development of leaf bulb, and then affect the yield and quality of Chinese cabbage. The development of Chinese cabbage is controlled by both gene and environment. The completion of genome sequencing of Chinese cabbage in 2011 [15] provided important reference information for bioinformatics analysis, genetic breeding, and key functional gene mining of Chinese cabbage gene family system at the whole genome level.

At present, multiple gene families of Chinese cabbage such as *HSF* [16], *AQP* [17], *TCP* [18], and *MYB* [19] have been identified by bioinformatics methods, and some genes have also been functionally verified. However, the identification and expression pattern response to various stresses of *SRO* gene families in Chinese cabbage have not been reported until now. Therefore, in this study, based on whole genome sequencing results, the members of *SRO* gene family in Chinese cabbage were identified via a bioinformatics analysis method, and subsequently the physical and chemical properties, evolutionary characteristics of its members, and protein structure were analyzed. Finally, the expression pattern of *BrSRO*s’ response to high temperature, low temperature, drought, and salt stress were set up via real-time quantification PCR methods. Our study provides a foundation for further research on the molecular mechanism of *SRO* gene mediating physiological growth process and stress response, and a significant basis for the genetic improvement of Chinese cabbage.

## 2. Result

### 2.1. Identification and Chromosomal Location of the SRO Family Genes in Chinese Cabbage

In this study, a total of 12 *BrSRO* genes were identified in the genome network of Chinese cabbage (Table 1). All genes were named respectively from *BrSRO1* to *BrSRO12* according to their position from the top to the bottom of Chinese cabbage chromosomes A02–A09. The number of amino acids of *BrSRO* genes is between 77–779 aa, with *BrSRO12* encoding the longest protein and highest molecular weight (85,523.47) and *BrSRO1* encoding the shortest protein and lowest molecular weight (8830.55). Furthermore, the isoelectric point changed from 6.02 (*BrSRO10*) to 9.6 (*BrSRO1*) and instability index changed from 33.9 (*BrSRO1*) to 59.1 (*BrSRO2*). *BrSRO1* has the largest fat index (100.13); the fat indexes of the rest *BrSRO* genes are between 61.69 and 88.16. In addition, the protein subcellular localization prediction showed that *BrSRO1* and *BraSRO9* proteins were predictably located in the chloroplast and nucleus. *BrSRO2*, *BrSRO4*, *BrSRO7*, and *BrSRO11* were predictably located in the chloroplast. The remaining genes were predicted to be located in the nucleus.

**Figure 1:**
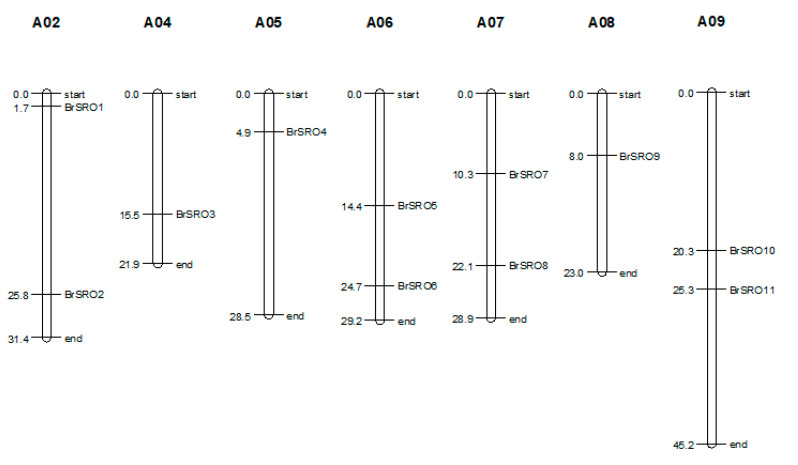
The chromosomal mapping analysis of the *SRO* gene family in Chinese cabbage. The chromosome number (A02–A09) is indicated at the top of each chromosome. The numbers on the left of each chromosome represent the initial position of each gene.

The identified 12 *SRO* genes in Chinese cabbage were mapped onto chromosomes or scaffolds. Among these, 11 genes (*BrSRO1-11*) were located in chromosomes, whereas the *BrSRO12* were distributed in unmapped scaffolds (Figure 1). In detail, the 11 predicted *BrSROs* were distributed unevenly across its 7 chromosomes. Each of chromosomes A02, 06, 07, and 09 harbored two *BrSRO* genes, and a single *BrSRO* gene was located in each of the chromosomes A04, 05, and 08.

### 2.2. Phylogenetic Analysis of the SRO Family Genes in Chinese Cabbage

**Figure 2:**
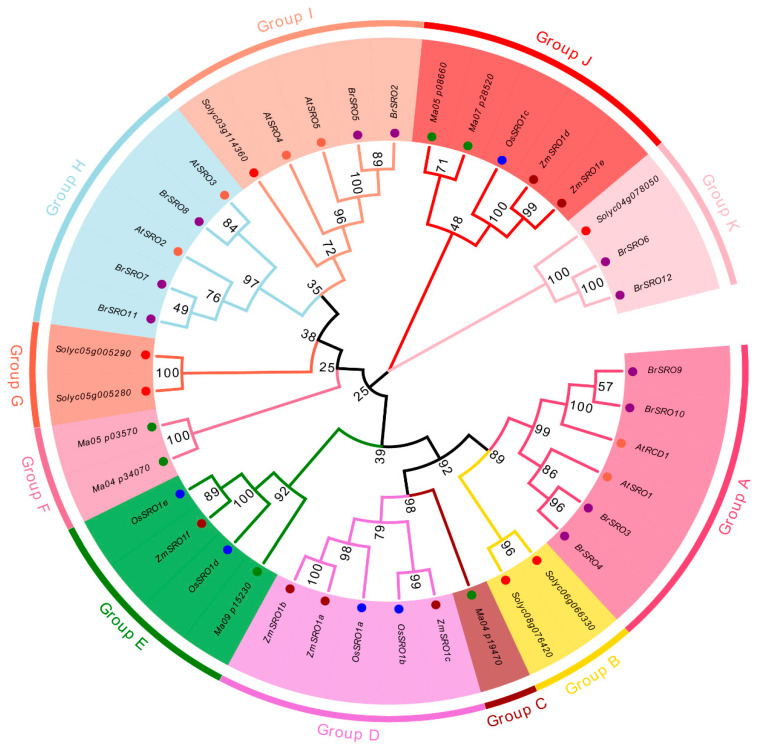
Phylogenetic tree of the similar to radical-induced cell death 1 (*SRO*) genes from *Arabidopsis thaliana* (At), *Solanum Lycopersicum* (Sl), *Brassica rapa* (Br), *O. sativa* (Os), *Zea mays* (Zm), *Musa acuminate* (Ma). In total, 6 AtSROs, 6 SlSROs, 11 BrSROs, 5 OsSROs, 6 ZmSROs, and 5 MaSROs were included. The phylogenetic tree was constructed for the SRO protein sequences in *Arabidopsis thaliana* (tomato), *Solanum Lycopersicum* (red), *Brassica rapa* (purple), *Oryza sativa* (blue), *Zea mays* (darkred) and *Musa acuminate* (green) using the Maximum-Likelihood method in MEGA 7.0. Bootstrap values from 1000 replicates are displayed at each node. The proteins on the tree can be divided into 11 groups from Group A to Group K, and the different groups are indicated by different colors.

The SRO proteins in Chinese cabbage were compared with other species to investigate the evolutionary relationships of SRO proteins. A phylogenetic tree was constructed on the basis of 40 putative nonredundant SRO protein sequences from six species, including *Arabidopsis*, banana, rice, *Solanum lycopersicum*, *Zea mays* and Chinese cabbage (Figure 2). All 40 SRO proteins were clustered into eleven groups (A–K), which consisted 6, 2, 1, 5, 4, 2, 2, 5, 5, 5, and 2 members, respectively. All *BrSROs* were clustered into Group A, H, I, and K, which indicated that the *SRO* of Chinese cabbage gene has higher homology with the *Arabidopsis* and tomato genes, compared with rice, maize and banana. The low bootstrap values in the tree are due to divergent SRO protein sequence among *Arabidopsis*, banana, rice, *Solanum lycopersicum*, *Zea mays*, and Chinese cabbage. This is not surprising, given that both *A. thaliana* and *B. rapa* belong to cruciferous plants, and the *SRO* genes in these two species were clustered together.

### 2.3. Gene Structure of the BrSRO Genes

**Figure 3:**
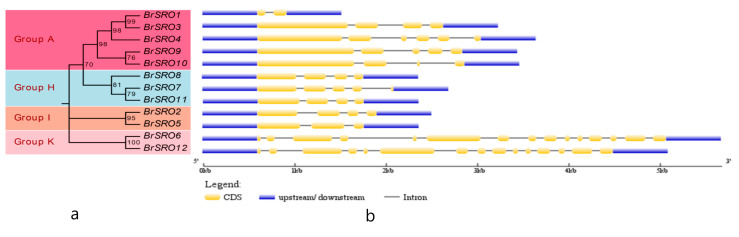
Compositions of introns and exons of *BrSRO* genes based on the phylogenetic relations. The amino acid sequences of the SRO proteins were aligned with ClustalX, and the phylogenetic tree was constructed using the neighbor joining method in MEGA 7.0 software (**a**). Each node is represented by a number that indicates the bootstrap value for 1000 replicates. The right side illustrates the exon-intron organization of the corresponding *SRO* genes. The exon and intron are represented by the yellow boxes and black lines, respectively. The scale bar represents 1 kb (**b**). The blue boxes represented upstream/downstream.

The predicted exon–intron structure was analyzed to gain an insight into the variation of the *SRO* genes in Chinese cabbage. On the basis of the evolutionary relationships of the Chinese cabbage phylogenetic tree (Figure 3a), the structure features were determined (Figure 3b). Phylogenetic analysis indicated that 12 *BrSRO* family members were divided into four groups (A, H, I, and K). All of the 12 *BrSRO* genes have complete gene structure. Interestingly, the exon-rich *BrSRO* genes containing 15 exons were clustered in group K, while the number of exons in the rest of groups ranged from 2 to 5, and the exon number of *BrSRO1* was the lowest. 

### 2.4. Conserved Motifs Analysis of BrSRO Proteins

**Figure 4:**
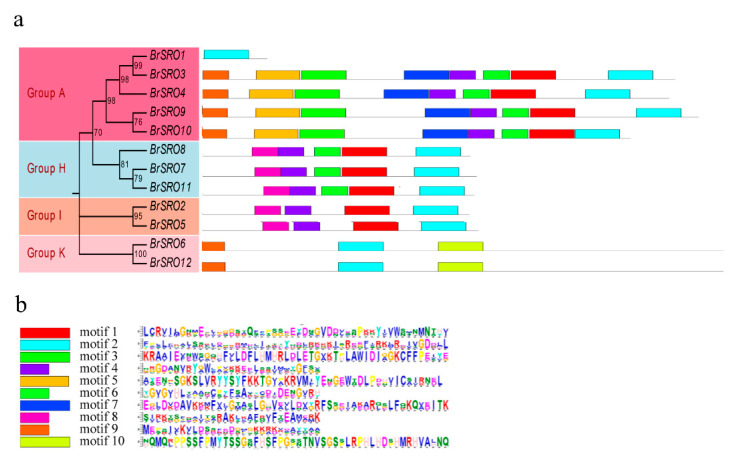
The conserved motifs of the BrSRO proteins based on the phylogenetic relationship. The BrSRO proteins phylogenetic relationship (**a**). The BrSRO proteins annotated with the MEME server (**b**). Distribution of the BrSRO conserved motifs in Chinese cabbage was analyzed by the online tool MEME. Ten motifs are marked by different colors.

The phylogenetic relationship and classification of *BrSROs* were further supported by motif analysis (Figure 4). Ten (10) conserved motifs of *BrSROs* were captured by motif analysis using MEME suite. All genes have the motifs (motif 1, 2, 3, 4, 5, 6, 7, and 9) in A group except for *BrSRO1*. The genes of the H group (*BrSRO7*, *BrSRO8*, and *BrSRO11*) have the same motifs, which are motif 1, 2, 4, 6, and 8. In addition, the genes in group I (*BrSRO2* and *BrSRO5*) have the same motifs, which are motif 1, 2, 4, and 8. There are only three motifs (motif 2, 9, and 10) in Group K. Interestingly, all 12 genes have motif 2, which indicates that motif 2 is relatively conservative.

### 2.5. Cis-Elements in the Promoters of BrSRO Genes

In order to study the response of *BrSRO* gene to various signal factors, we searched 2 kb sequences upstream of the start codon of *BrSROs* family for elements related to stress response. There are many light signal elements (MRE, box-4, TCT motif, etc.), hormone and stress response elements. The cis-acting elements related to hormones and stress response in *BrSRO* gene promoter were analyzed and illustrated. It can be seen from the table that *BrSRO* gene promoter contains 12 cis-elements that respond to hormones and stress. Interestingly, 12 *BrSRO* genes include at least one of the 12 predicted types of cis-elements in their promoter regions (Table 2). 10 *BrSRO* genes contain the ABRE cis-element; only two genes (*BrSRO1* and *BrSRO9*) lack it. There are more MeJA response elements (CGTCA-motif, TGACG-motif) in the *BrSRO* genes than other cis-elements. MBS is located in *BrSRO1*, *3*, *4*, *7*, *8*, *11*, and *12*. All genes except *BrSRO1*, *4*, *5*, *8*, and *10* have TATC-motif/P-box, indicating they are related to GA response. Only *BrSRO3*, *4*, *5*, and *7* have LTR and only two genes, namely, *BrSRO2* and *BrSRO8*, contain the TC-rich repeats cis-element in their promoter regions. These results suggest that SRO family may play a crucial role in the growth and development of Chinese cabbage, as well as in various hormones and stress.

### 2.6. Relative Expression of 12 BrSRO Genes

**Figure 5:**
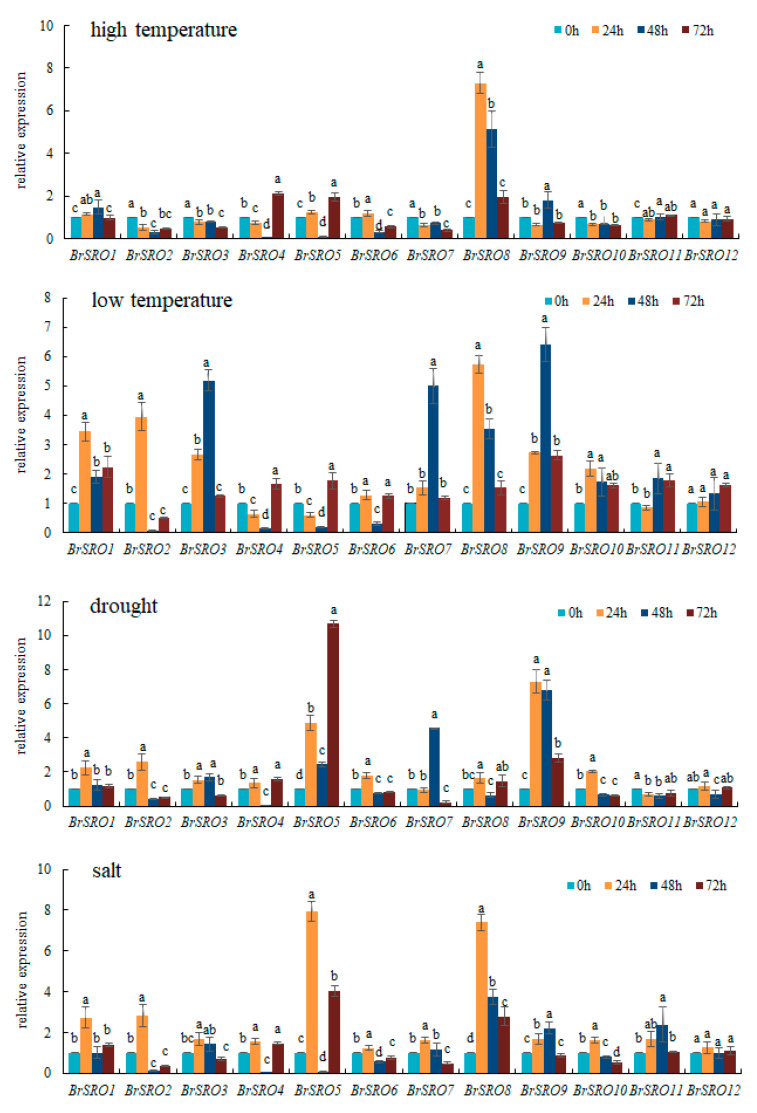
Expression profiles of 12 *BrSRO* genes in response to high temperature treatment, low-temperature treatment, drought treatment, and salt treatment. Quantitative reverse transcription polymerase chain reaction (qRT-PCR) analyses were used to assess the transcript levels of *BrSROs* in leaves sampled at 24 h, 48 h and 72 h after high temperature, low-temperature treatment, drought, and salt treatment in Chinese cabbage seedlings. 0 h as control. Three sets of repeats are set for each process. Error bars indicate standard deviations of three replicates and different letters describe significant differences at *p* ≤ 0.05 level among different time points within the same gene.

Using qRT-PCR, the relative expression levels of *BrSRO* genes in leaf were analyzed under abiotic stresses for 24 h, 48 h, and 72 h. The results showed that the expression of *BrSROs* responded differently to various abiotic stresses. Under high temperature stress, the relative expression levels of *BrSRO1*, *5*, *6*, and *8* genes were up-regulated and the rest of genes was down-regulated at 24 h. The relative expression levels of *BrSRO1*, *8*, and *9* genes were up-regulated and *BrSRO4* and *BrSRO5* were down-regulated at 48 h, while *BrSRO4*, *5* and *BrSRO8* were up-regulated at 72 h. Interestingly, the relative expression level of *BrSRO8* was significantly up-regulated at three time points and reached the highest level at 24 h (Figure 5). Under low temperature, the relative expression levels of *BrSRO1*, *3*, *7*, *8*, *9*, and *12* genes were up-regulated at three time points and the up-regulated amplitudes of different genes were different at different time points (Figure 5). Under drought stress, the relative expression levels of *BrSRO1*, *5*, and *9* genes were up-regulated at three points and the relative expression level of *BrSRO5* reached the highest level at 72 h while *BrSRO9* reached the highest level at 24 h and 48 h (Figure 5). Under 2%NaCl treatment, the relative expression level of all the *BrSRO* genes were up-regulated at 24 h, moreover, *BrSRO5* and *BrSRO8* were significantly up-regulated and about 7.5 times the control. At 48 h, the *BrSRO1*, *3*, *7*, *8*, *9*, and *11* were up-regulated while *BrSRO2*, *3*, *4*, and *5* were reached lowest for 48 h. Only *BrSRO1*, *8* and *12* up-regulated at 24 h, 48 h, and 72 h (Figure 5). Thus, it could be seen that the up-regulation of *BrSRO1* and *8* genes were significant under all treatments, while the up-regulation of *BrSRO9* was significant under drought, low temperature, and salt stresses. The expression of *BrSRO12* was not significantly up-regulated or down-regulated in all treatments compared with the control. *BrSRO5* gene was significantly up-regulated under drought and salt treatments, and *BrSRO7* gene was significantly up-regulated under drought and low-temperature treatments. The above candidate genes (*BrSRO1*, *5*, *7*, *8*, and *9*) were used for functional analyses in the succeeding experiment.

## 3. Discussion

The SRO protein family is highly conserved and found in all land plant species [5]. Several *SROs* have been identified as involved in plant development and stresses response. However, the family members and functions of *SROs* are largely unknown in Chinese cabbage. The exact biochemical functions of the SRO proteins remain unknown. The *SROs* are characterized by the plant-specific domain architecture which contains a poly(ADP-ribose) polymerase catalytic (PARP, PF00644) and a C-terminal RCD1-SRO-TAF4 (RST, PF12174) domain [5]. In addition to these two domains, some SRO proteins have an N-terminal WWE domain (PF02825). The RST domain is plant-specific and present in SROs and TAF4 proteins. Previous studies have demonstrated that PARP-RST domains are specific to plants, while WWE-PARP domains are widely conserved in organisms even as distantly related as humans [20,21]. The RST domain is essential for the interaction between RCD1 and other TFs [3]. PARPs are a class of enzymes that are involved in many biological processes, including DNA damage repair, transcription, cell death pathways, and chromatin modification/remodeling [22]. In this study, a total of 12 *BrSROs* genes were identified from the Chinese cabbage genome and named *BrSRO1*–*BrSRO12*, according to chromosome location. Only two genes, *BrSRO4* and *BrSRO9*, were identified as having the WWE domain, whereas the rest of *BrSROs* only have the RST domain and PARP domain, lacking WWE domain. From the analysis of physicochemical properties of protein, the number and molecular weight of amino acids are quite different between *BrSRO1* and *BrSRO12*, which indicates that there are some differences in their structure and function. Phylogenetic tree analysis showed that SRO proteins of Chinese cabbage and *Arabidopsis thaliana* were highly similar, and their genetic relationships were also similar, and we can infer that there is functional similarity. The study of exons and introns is helpful to understand the differences of gene structure and function [23]. The number of *BrSRO* exons in the same group was very close, so most genes showed conservative gene structure, which supported a close evolutionary relationship [23]. Interestingly, the K group was located in the exon-rich region. It was proposed that the rates of intron creation are higher during earlier periods of plant evolution [24]. Additionally, the rate of intron loss is greater than the rate of intron gain after segmental duplication. Thus, it is possible that the group K may represent the original genes of *SRO* family [24]. Motif analysis further demonstrated the structural similarity of A, H, I, and K groups. All genes have motif 2, indicating that RST domain exists in motif 2. All genes contain cis-acting elements of light response. There are more gibberellin response elements, and methyl jasmonate response elements. Methyl jasmonate elements are important phytohormones that mediate plant development and defense mechanisms against biotic (i.e., necrotrophic pathogen infection and herbivorous insect attack) and abiotic (i.e., mechanical wounding) stress [25]. Methyl jasmonate can also be used as the core signal factor of plant resistance to insect invasion [26]. Salicylic acid (SA) is an important signaling molecule for plants to cope with biotic or abiotic stress [12]. Gibberellins are phytohormones that regulate multiple developmental processes, such as seed germination, stem elongation, flowering, and fruit development [27]. Many cis elements related to abscisic acid and drought stress response were found in the promoter region of *BrSRO* gene, which indicated that *BrSRO* gene family might respond to drought stress through the hormone signal transduction pathway. 

The *SRO* family not only affects plants growth and development, but also affects their response to various stresses. The *SRO* family has proven to be able to respond to abiotic stress in many plants. For example, the relative expression level of *OsSRO1c* was significantly up-regulated under ABA and JA treatments. *Ta*-*SRO1* can regulate the oxygen content in wheat. Chemical reduction balance was used to improve the tolerance to drought, high salt, and H_2_O_2_ stress [1]. The expression level of *MdSRO4* in apples treated with 100 μmol L^−1^ ABA and 4 °C were 14 and 37 times higher than that of ABA and 4 °C, respectively. Under 20% polyethylene glycol (PEG) treatment, the relative expression levels of *MdRCD1*, *MdSRO2*, and *MdSRO3* were up-regulated by 18, 17, and 14 times compared with that of *MdSRO4*, respectively, indicating that *MdSRO4* could respond to ABA and chilling stress, *MdRCD1*, *MdSRO2*, and *MdSRO3* could respond to drought stress [13]. In this study, the expression levels of *BrSRO* genes in leaves were analyzed under abiotic stresses for 24 h, 48 h, and 72 h. Our results showed that the responses of *BrSROs* were different among heat, low temperature, drought, and salt stresses. *BrSRO8* is sensitive to high temperature, and the expression of *BrSRO1*, *3*, *7*, *8*, and *9* was higher under low temperature treatment. The response to drought stress was *BrSRO1*, *5*, and *9*, and to NaCl stress was *BrSRO7* and *BrSRO8*. The expression level changed with the time of treatment; it may be that plants regulate themselves to resist changes in the external environment. Interestingly, the expression levels of all 12 genes were up-regulated after 24 h salt treatment, indicating all the genes responded to salt stress at 24 h. Excess salts in soils cause growth arrest, molecular damage, and even the death of many of the salt-sensitive crop species that are grown today [28,29]. Thus it can be predicted that the *BrSRO* genes family may play an important role in the resistance to salt stress. Two *SRO* genes, *GHSRO04* (Gen bank accession number kr534896) and *GHSRO08* (Gen bank accession number kr534895) have been cloned from upland cotton. The two genes were induced to express by high salt and drought, indicating that *SRO* plays an important role in regulating the growth and development of cotton under pathogen attack, salt, and drought stresses, and has potential utilization value for the genetic improvement of cotton germplasm [30]. Whether the function of *SRO* genes in Chinese cabbage work under biotic and abiotic stresses or not, the candidate genes with higher expression levels (*BrSRO1*, *5*, *7*, *8*, and *9*) at three time points under abiotic stresses were selected to further verify their functions in the future.

## 4. Materials and Methods

### 4.1. Identification and Sequence Analysis of SRO Genes in Chinese Cabbage

Six known ID of *Arabidopsis thaliana SRO* genes [12] were put into *Arabidopsis* genome database (TAIR) [31] to obtain their protein sequences. Using *Arabidopsis* SRO protein sequences as probes, the candidate members of Chinese cabbage SRO family were searched and the coding sequences (CDS) and amino acid sequences of the *B. rapa* SRO genes were downloaded from the Brassica database [32]. The banana SRO genes and protein sequences were downloaded from the Banana Genome Hub [33], the rice SRO genes and protein sequences from the Rice Genome Annotation Project [34], and the website of Phytozome [35] was used to search for the SROs from *Solanum lycopersicum* and *Zea mays*. The candidate sequences with conservative domains of PARP (PS51059) and RST (PF12174) were then inspected using the SMART program [36]. Subsequently, the Prot-Param tool [37] was used to analyze the physicochemical parameters (i.e., length, molecular weight, and isoelectric point) of the SRO proteins. Subcellular localization prediction was carried out with the Plant-mPLoc [38].

### 4.2. Phylogenetic Analysis of SRO Genes in Chinese Cabbage

The phylogenetic tree was constructed with MEGA 7.0 (https://www.megasoftware.net/home) [39] on the basis of alignment with the amino acid sequences of the *BrSRO* proteins using the neighbor-joining method [40] with 1000 bootstrap replicates [41]. 

### 4.3. Gene Structure and Conserved Motifs Analysis of BrSROs

The distribution of the conserved motifs based on amino acid sequence was conducted with the online MEME program [42] and the MEME search was carried out with the following parameters: maximum number of motifs set at 10, a minimum width of 6 and a maximum width of 50. The other parameters were set as default. The exon-intron structure of each *BrSRO* was determined by aligning the full-length cDNA sequence with the genomic DNA sequence. The schematic structure of each *BrSRO* was constructed using the Gene Structure Display Server (GSDS 2.0) (http://gsds.cbi.pku.edu.cn) [43].

### 4.4. Chromosomal Distribution and Cis-Element Analyses of SRO Genes in Chinese Cabbage

The information about chromosomal distribution was obtained from the Chinese cabbage genome database [32], and the chromosomal location of *BrSRO* genes was illustrated from top to bottom concerning their position in the genome annotation using Mapchart [44]. For identification of cis-elements located at the promoter regions of *SRO* genes, the 2000 bp genomic DNA sequences upstream before the initiation codon (ATG) of each *BrSRO* gene were downloaded from the Chinese cabbage genome database. The PlantCARE database [45] was utilized to search the cis-regulatory elements in promoter regions of Chinese cabbage genes. 

### 4.5. Plant Materials, Growth Conditions and Treatments

In this study, the plants used for expression analysis were sampled from the “furui” Chinese cabbage seedlings. The seeds were soaked in water for 2 h then placed on moist filter paper in petri dish, and finally kept in the dark to germinate at 25 °C for 16 h. After germination, uniformly geminated seeds were sown in 50-hole tray filled with substrate and then put in an artificial climate chamber. The growth condition of the artificial climate chamber was as follows: photoperiod 12 h/12 h, temperature 25 °C/18 °C (day/night), relative humidity 80%, light intensity 250 μmol·m^−2^·s^−1^. After sowing for 23 days, the uniform seedlings were selected and treated with low temperature 10 °C/5 °C (day/night), high temperature 35 °C/20 °C (day/night), 2% NaCl solution, and under natural drought conditions for 0 h, 24 h, 48 h, and 72 h. The leaves treated with high temperature, low temperature, salt and drought stress for 0 h, 24 h, 48 h and 72 h were sampled, which was frozen with liquid nitrogen and stored at −80 °C for the following experiment.

### 4.6. RNA Isolation and qRT-PCR Analysis

Total RNA was extracted from leaf tissues by using the Plant RNA Extraction Kit (Takara, Kusatsu, Japan). The first-strand cDNA fragment was synthesized from total RNA by using the Prime Script^®^ RT Reagent kit (Takara, Kusatsu, Japan). The reverse transcripts were preserved at 20 °C for the following PCR amplification. The CDS sequences of *BrSRO* genes were input into the homepage of Shanghai biology company (Shanghai, China) for online primer design (as shown in Table 3), and then the primer sequences were synthesized. The *actin* gene was used for internal reference. The amplification system contained 2 μL cDNA, upstream primers 0.6 μL, downstream primers 0.6 μL, Rox 0.4 μL, SYBR 10 μL, reaction mix 6.4 μL, and ddH_2_O 20 μL. The PCR cycling conditions included an initial polymerase activation step of 95 °C for 15 min, followed by 40 cycles of 95 °C for 10 s, and 60 °C for 30 s. Three biological replications for each sample were done. The relative expression levels of the *BrSRO* gene are represented in the form of relative changes by the 2^−∆∆Ct^ method [46]. Three biological replicates were carried out and the significance was determined with SPSS software. (SPSS 17.0, IBM, Chicago, IL, USA) (*p* ≤ 0.05)

## Figures and Tables

**Table 1:** Physiochemical characteristics of identified BrSRO genes.

Gene Name	Gene ID	Chromosome Location	Protein Length (aa)	Molecular Weight (kd)	PI	Total Number of Atoms	INSTABILITY Index	Fat Index	Predicted Subcellular Localization
*BrSRO1*	*Bra033139*	Chromosome A02: 16,846,412–16,846,733	77	8830.55	9.6	1272	33.9	100.13	Chloroplast. Nucleus.
*BrSRO2*	*Bra029254*	Chromosome A02: 26,296,663–26,297,969	303	34,150.08	8.68	4793	59.1	82.71	Chloroplast.
*BrSRO3*	*Bra017317*	Chromosome A04: 15,393,395–15,395,430	530	58,637.64	6.99	8192	34.19	80.55	Nucleus.
*BrSRO4*	*Bra005336*	Chromosome A05: 4,905,877–4,908,322	524	58,577.61	6.1	8178	37.71	80.29	Chloroplast.
*BrSRO5*	*Bra010096*	Chromosome A06: 19,383,958–19,385,124	313	34,876.77	8.59	4889	57.49	80	Nucleus.
*BrSRO6*	*Bra033662*	Chromosome A06: 25,753,358–25,757,833	771	85,056.17	9.07	11,856	57.48	63.97	Nucleus.
*BrSRO7*	*Bra012380*	Chromosome A07: 8,098,261–8,099,752	310	34,575.22	8.86	4865	45.5	85.29	Chloroplast.
*BrSRO8*	*Bra016219*	Chromosome A07: 18,821,147–18,822,313	304	33,789.47	8.15	4744	39	88.16	Chloroplast.
*BrSRO9*	*Bra035511*	Chromosome A08: 7,983,100–7,985,345	558	62,697.49	6.59	8754	37.36	79	Chloroplast. Nucleus.
*BrSRO10*	*Bra023252*	Chromosome A09: 20,223,502–20,225,770	482	54,230.68	6.02	7557	42.34	78.28	Nucleus.
*BrSRO11*	*Bra024609*	Chromosome A09: 24,077,869–24,079,029	308	33,418.73	6.19	4682	40.86	84.84	Chloroplast.
*BrSRO12*	*Bra035961*	Scaffold000111: 11,933–15,826	779	85,523.47	8.98	11,902	55.21	61.69	Nucleus.

**Table 2:** Putative cis-elements existed in the 2 kb upstream region of *BrSRO* gene family.

Gene	Hormonal Response Cis-Elements								Stress Response Cis-Elements			
	Abscisic Acid Response Element	Methyl Jasmonate Response Element		Salicylic Acid Response Element	Auxin Response Element	Gibberellin Response Element			Anaerobic Induction Response Element	Drought Response Element	Low-Temperature Response Element	Defense and Stress Response Element
	ABRE	CGTCA-Motif	TGACG-Motif	TCA-Element	TGA-Element	GARE-Motif	TATC-Box	P-Box	ARE	MBS	LTR	TC-Rich Repeats
*BrSRO1*	0	3	3	0	0	1	0	0	2	3	0	0
*BrSRO2*	4	2	2	0	3	0	2	1	3	0	0	1
*BrSRO3*	1	0	0	0	0	0	1	1	4	4	1	0
*BrSRO4*	5	0	0	1	2	0	0	0	4	1	1	0
*BrSRO5*	5	0	0	0	2	0	0	0	0	1	1	0
*BrSRO6*	1	2	2	0	2	0	0	0	5	0	0	0
*BrSRO7*	2	0	0	0	1	0	0	1	1	1	1	0
*BrSRO8*	3	5	2	0	0	0	0	0	2	1	0	1
*BrSRO9*	0	3	3	0	1	1	0	0	4	2	0	0
*BrSRO10*	1	4	5	1	1	1	0	3	0	0	1	0
*BrSRO11*	3	3	3	0	0	0	0	1	0	0	0	0
*BrSRO12*	5	4	4	1	1	0	0	2	0	2	0	0

**Table 3:** The sequences of primers used for qRT-PCR.

Gene Name	Forward Primer Sequence (5′-3′)	Reverse Primer Sequence (5′-3′)
*BrSRO01*	AAGCTGAGGATGATTGTTGGAGA	CAAAGCAGTGTGTGGTAAGCG
*BrSRO02*	GGGTTTGCCGCCGTTGGATC	TTTGCCGCCGCCTTCTTCAC
*BrSRO03*	AAGCCTGCTGAGGAGGAAGACC	CGACGCCACCTGAAAACCTATACG
*BrSRO04*	GAACTCACGGCTCACCTTGGAAG	GAGCAGAGGGTAAGGCATCAAAGC
*BrSRO05*	AGCTGCGGAGTCGGAAGATGG	CCTCGTGGAACAACCTCAGACTTC
*BrSRO06*	AATGAATGCTCGTGGTCCGTTGG	GCTTGGTGGTGGCGGTGAAG
*BrSRO07*	GCGATCACCACGAGAGCCAAG	AGCCAGCGTACCAACCGTATTTG
*BrSRO08*	GCGGAGGCTATGAAGAGGAAGAAC	CGACCTCGCTGCTGCTAAACC
*BrSRO09*	CACCAAACCCGCAGACCCAAG	TGACCAGCGACTTCCCAGAGC
*BrSRO10*	TCTGGTGTCAAGCCTGCTGGAG	CGAGCTTCCGCAATCTCACTGG
*BrSRO11*	GCGGTTGTGTCAGTGCTGTCC	GCCACTTGTCTCATCTTCCGAACC
*BrSRO12*	GTGTGGAAGAAAGGATGCGAGGAC	CGTTGATTTGCTGCCGAACATCTG
*actin*	CCAGGAATCGCTGACCGTAT	CTGTTGGAAAGTGCTGAGGGA
